# Laboratory Diagnosis of Scabies Using a Simple Saline Mount: A Clinical Microbiologist's Report

**DOI:** 10.7759/cureus.1102

**Published:** 2017-03-19

**Authors:** Venkataramana Kandi

**Affiliations:** 1 Department of Microbiology, Prathima Institute of Medical Sciences

**Keywords:** scabies, sarcoptes scabiei, mite, skin diseases, simple saline mount, skin scrapings

## Abstract

Scabies is a skin condition caused by infestation of the mite Sarcoptes scabiei. Sarcoptes scabiei var. hominis is an arthropod member belonging to the order Acarina. Scabies is present throughout the world and is prevalent in all age groups, mostly involving people frequently in contact with animals, children, women, and elderly people. Predisposing factors for scabies include individuals with immunosuppressive conditions and people residing under low socioeconomic conditions. Skin lesions that occur during mite infestation closely resemble dermatological disorders caused by microbes, including fungi, parasites, and viruses. Laboratory diagnosis of scabies greatly relies on an accurate clinical suspicion and the demonstration of mites in the skin scrapings could be used for confirmation. It should be noted that a greater understanding between a clinician or a dermatologist and a clinical microbiologist is required to successfully diagnose scabies. This report details an easily performed, cost-effective method, the simple saline mount, that a clinical microbiology laboratory should follow to successfully identify mites in skin scrapings.

## Introduction

Parasitic infestation is not uncommon in both humans and animals. Infestations by insects and fly larvae have always been ignored, majorly due to their scattered and infrequent reports in the literature [1-2]. This doesn’t undermine their relevance in causing both infections as well as infestation in both human and animals. Scabies is a skin condition that results due to infestation of a mite, Sarcoptes scabiei. Scabies, the mite infestation in animals, is commonly called mange. Scabies is an ectoparasitic infestation, a skin disease, prevalent throughout the world with increased occurrence in developing third world and socio-economically weaker countries [3-4].

Clinically, scabies has been found to present in three different forms including the classic scabies, nodular scabies, and the more severe, and highly contagious crusted scabies also known as Norwegian scabies. Sarcoptes scabiei is an obligate ectoparasite residing in the dermis, and epidermis of skin of both human and animals. It is an arthropod member belonging to the class Arachnida, order Astigmata, and family Sarcoptidae. The infestation starts with invasion of female mites into the stratum corneum of the host after which it lays eggs, later developing into larvae, nymph (protonymph and tritonymph), and adults. The number of infesting mites usually depends on the immunological condition of the host and the extent of spread. Although the mites do not fly, scabies easily spreads from one person to the other person by ordinary skin contact, and isolation of the patients is required to stop the spread, especially in the hospital environment. Laboratory diagnosis of scabies is complex, and most often, its diagnosis is missed due to lack of data on its prevalence and also due to the dermatological manifestations being similar to many other skin diseases. Although routine microscopic examination of skin scrapings for the presence of mites is considered as the gold standard for the diagnosis of scabies, the procedural errors and lack of communication between a dermatologist and microbiologists will result in false negative reports. A previous study has observed that a combination of dermatoscopy and light microscopic methods could improve scabies diagnosis and that there is need of a low cost, easily performed, and accurate technique [5]. This technical report describes the experience of a clinical microbiologist with the use of a simple saline mount of skin scrapings in the diagnosis of scabies.

## Technical report

Patients suffering from various skin disorders usually present themselves at the department of dermatology [5]. Dermatological manifestations can result from various causes that include common diaper rash, chemical-induced dermatitis, immunological conditions, genetic causes, and drug reactions [6]. Infections with bacteria, viruses, parasites, and fungi may also present with similar skin lesions. Dermatologists usually perform a clinical diagnosis, identify the specific areas of skin lesions and collect skin scrapings or biopsies for further confirmation of pathological and microbiological causes. It was previously reported that there is a 45% chance of misdiagnosing scabies with other skin conditions [5].

Clinical microbiologists most often receive a sample of skin scrapings in a dry, sterile test-tube or in a clean paper wrap. Most skin scrapings received in a clinical microbiology laboratory are processed generally for the presence of either bacteria or fungi and very rarely are received to screen for the presence of parasites and other microbes. 

**Figure 1:**
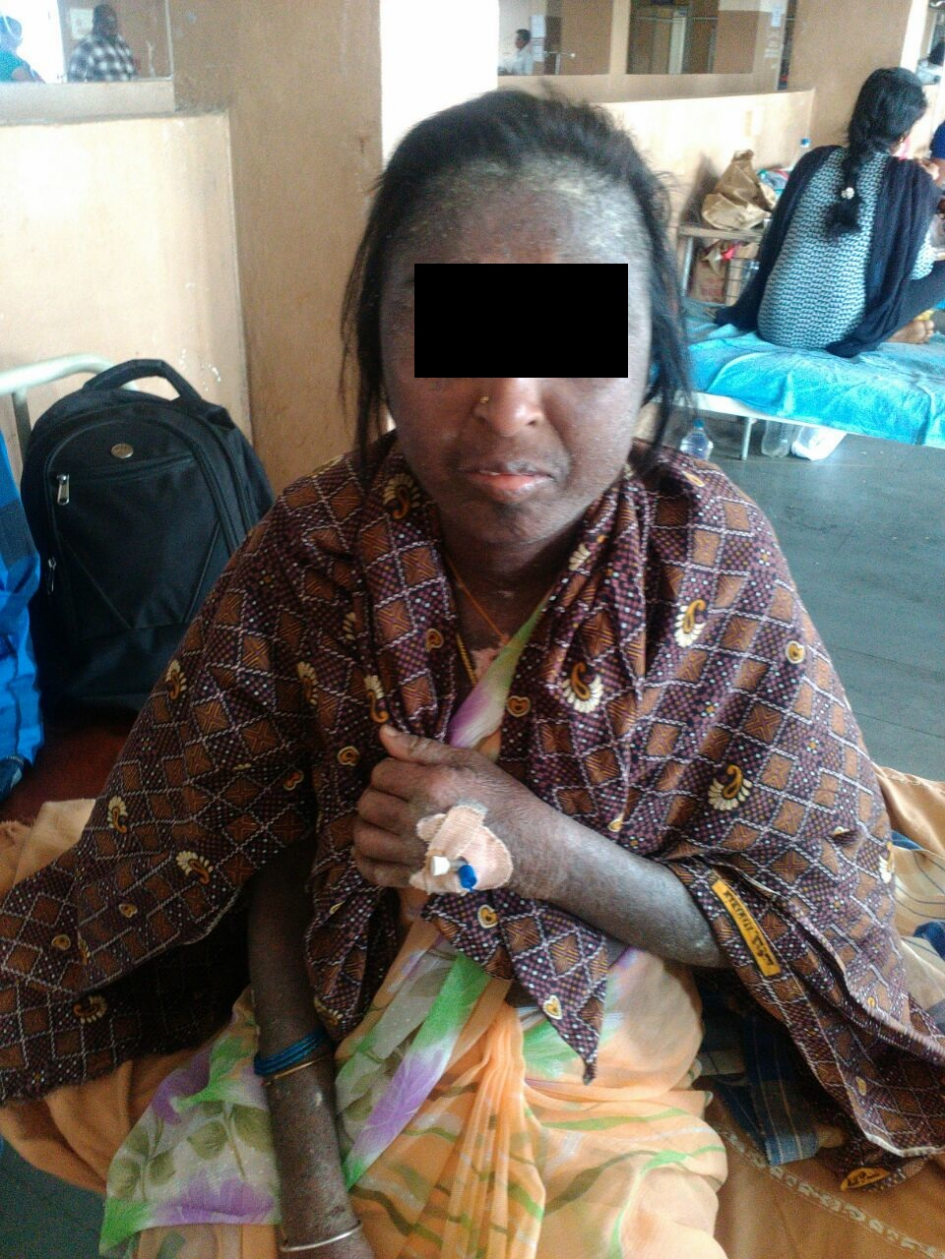
Mite-infested patient showing disseminated hyperpigmented and scaly skin lesions

In the present case, the patient's skin scrapings were sent to the laboratory asking for a potassium hydroxide (KOH) mount report with a provisional diagnosis of scabies and to rule out fungal infection, if any. The patient presented with extensive hyperpigmented lesions with scaling spread throughout the body, including the face, hands, and the feet, as shown in Figure 1.

**Figure 2:**
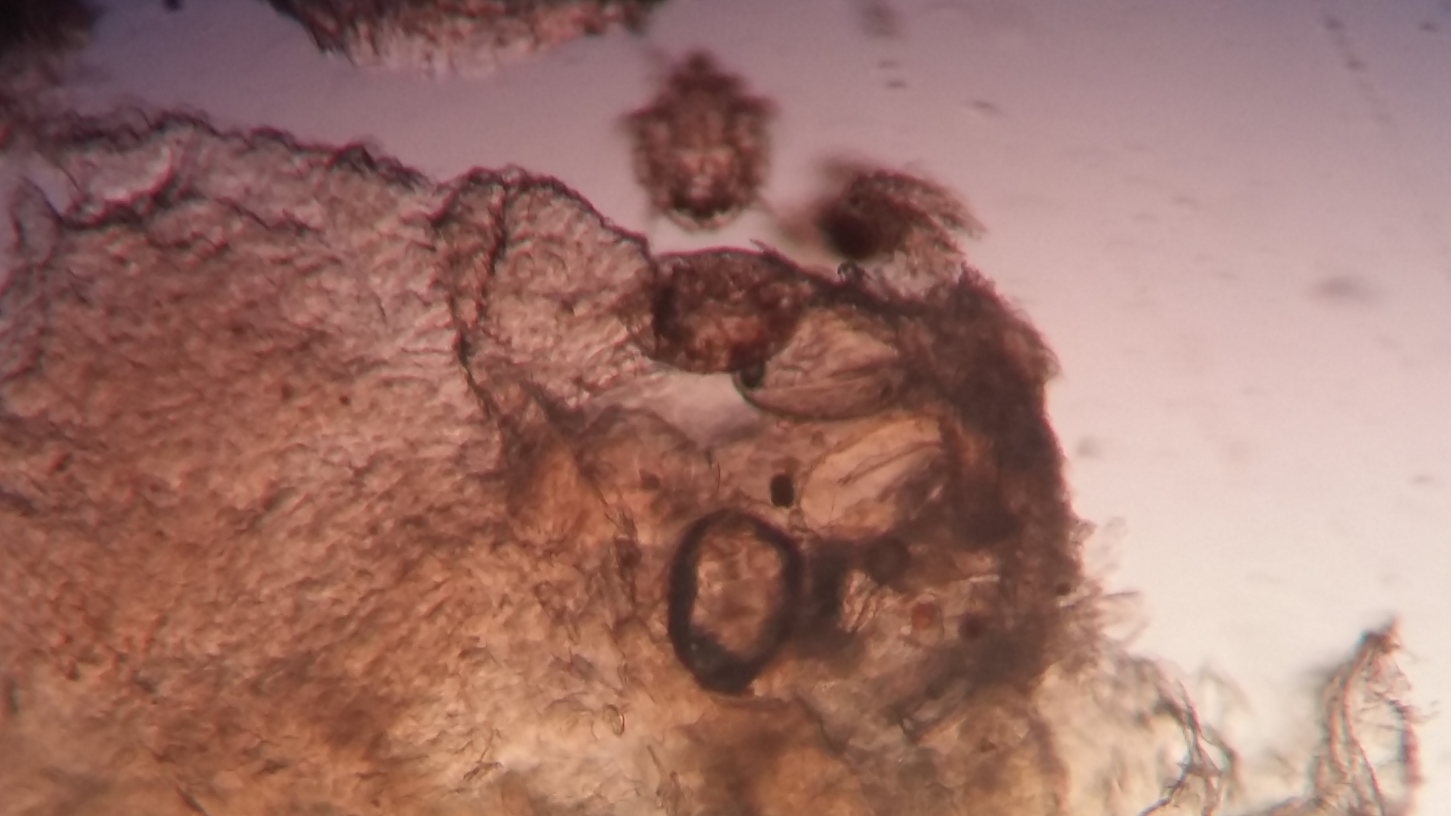
KOH preparation of skin scrapings reveal the presence of larval stages of mites within the skin

A routine KOH mount was performed by the laboratory technicians, where the skin scrapings were placed on a slide in a drop of 10% KOH and, after about 30 minutes, was observed under a low power (10X) and high power (40X) objective of a compound microscope for the presence of fungal elements. The KOH mount was initially screened by three microbiologists giving a negative result for fungal elements. None of them could recognize the presence of mites in the KOH mount. Not being able to recognize the presence of mite-like structures could be due to lack of a previous experience of seeing them and also could be attributed to the immobilized mites (due to the effect of KOH) masked under the similarly coloured skin as observed in Figure 2.

Simple saline mount

The skin scrapings were later treated with normal saline in a test-tube, and after about an hour, a simple wet mount was prepared. A drop of the sample was placed on a clean and grease-free slide, a coverslip was then mounted on it, and was observed under low power (10X) and high power (40X) objective of a compound microscope. Under 10X objective, again nothing was observed initially. However, when observing under 40X, slowly moving objects within the skin were noted. Later on, with keen observation, and due to the treatment of the skin with the saline for about an hour, a mite was observed to move out of the skin as shown in this Video 1.

**Video 1:** Live and motile mites wandering through skin scrapings as observed in a simple wet mount preparation

**Figure 3:**
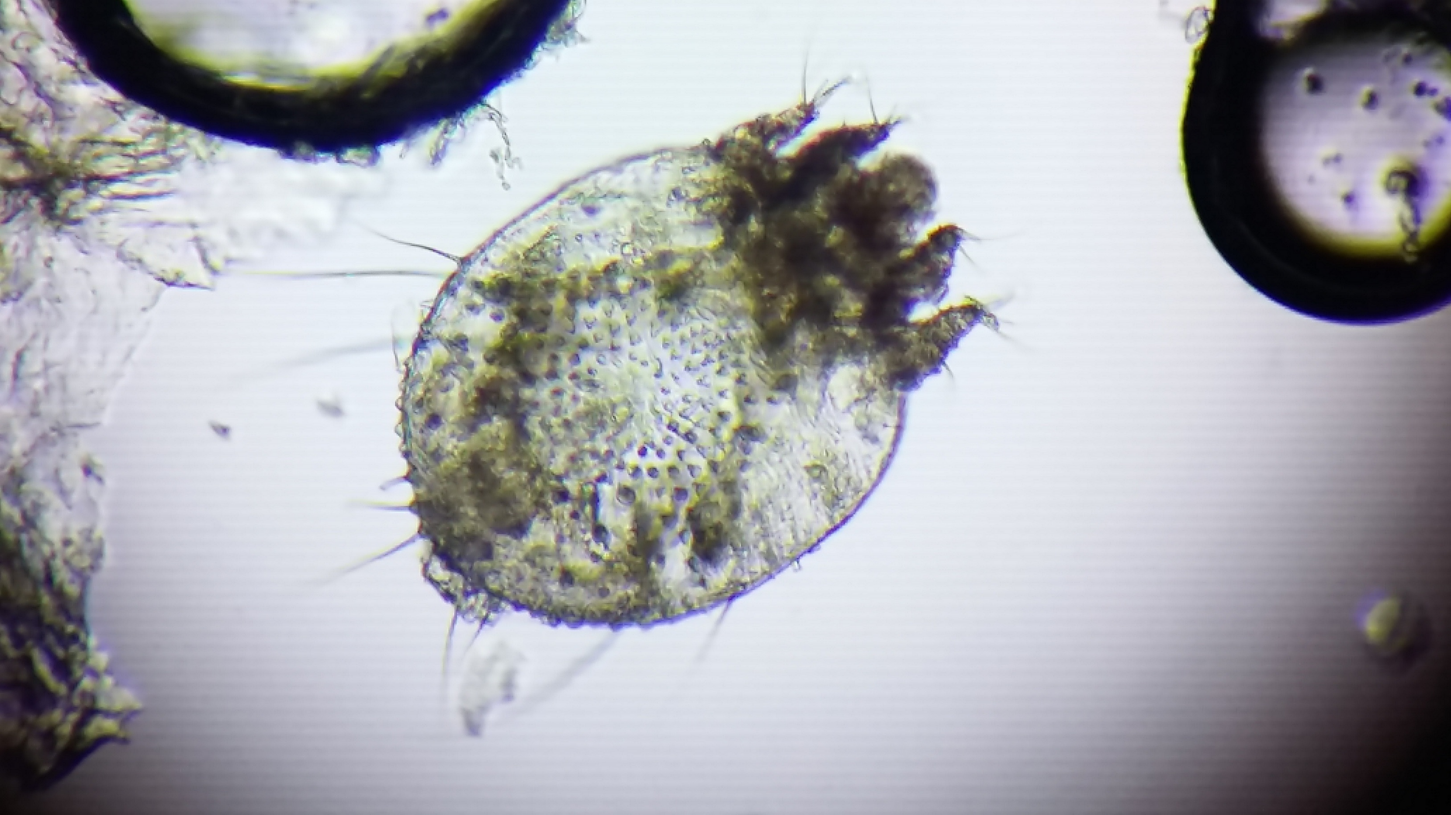
An adult mite as viewed after a simple saline wet mount

The mite could not survive, and after the mount had dried, a clear picture of it was seen as shown in Figure 3. 

## Discussion

Scabies is frequently reported among animals, including dogs, pigs, and other domestic and wild mammals. Sarcoptesmange, as it is commonly called in animals, presents with papules, pustules, erythema, pruritus, alopecia, hyperpigmentation, and crusting, depending on the severity of infestation. The Sarcoptes scabiei mite has been noted to survive in infested animals for up to 10 years if not diagnosed and treated. Infested animals pose a potential threat to humans and can result in human-to-human transmission. 

Scabies is now considered as a neglected disease, an ectoparasitic infestation in humans caused by mite species [7-8]. Observing the mites, their eggs, and pellets (faecal material/scybala) under a light microscope is considered to be the gold standard in the laboratory diagnosis of scabies [9]. A routine examination of skin scrapings could be negative, which does not rule out mite infestation [10]. From India, there are fewer reports on scabies, which could be attributed to lack of epidemiological data and failure in clinical suspicion, combined with the use of inaccurate methods in laboratory diagnosis [11]. 

It should be noted that a delay in the laboratory diagnosis in a hospitalized patient could pose a threat of infestation among other patients and to the healthcare workers as noted by previous studies [12-15]. There was also a recent report of an outbreak and spread of scabies in residential care centres, emphasizing the importance of knowledge and practice in the diagnosis and management of scabies [16].

Owing to the morbidity it can cause in different age groups, especially in children, as well as the contagious nature, it becomes very important to educate people about the significance of mite infestation, the potential predisposing factors, symptoms, and laboratory diagnosis [17-18]. Identification of scabies and differentiating it from other skin conditions could be important in making an accurate choice of treatment and in the better management of patients, as observed in a recent study by Tasani, et al. [19].

Laboratory diagnosis of scabies has moved way ahead from using traditional light microscopic techniques to improved methods, including the epiluminescence microscopy, the enzyme-linked immunosorbent assay (ELISA) for the detection of antigens and antibodies, and the development of conventional and real-time quantitative polymerase chain (qPCR) assay [20].

## Conclusions

An increased awareness of the occurrence of scabies and a strong clinical suspicion by a dermatologist are prerequisites for a laboratory diagnosis of scabies. Considering the fact that a routine fungal mount with KOH may immobilize or kill the mites, making it difficult to detect them, clinical microbiologists should process skin scrapings with a simple saline mount as well to improve the chances of finding the mites. Although there has been a great improvement in the knowledge of scabies, further research on the epidemiology, effective diagnosis, treatment, management, and prevention of scabies is required.
